# Potential of blood-based biomarker approaches in endometrium and breast cancer: a case-control comparison study

**DOI:** 10.1007/s00404-022-06482-8

**Published:** 2022-03-13

**Authors:** Anne Schuhn, Tania Witte Tobar, Aoife Ward Gahlawat, Jana Hauke, Lukas Baumann, Jürgen G. Okun, Juliane Nees

**Affiliations:** 1 Department of Gynecology and Obstetrics, University Hospital Heidelberg, Im Neuenheimer Feld 440, 69120 Heidelberg, Germany; 2 Department of General Pediatrics, Division of Neuropediatrics and Metabolic Medicine, Center for Pediatric and Adolescent Medicine, University Hospital Heidelberg, Im Neuenheimer Feld 430, 69120 Heidelberg, Germany; 3 Institute of Medical Biometry, University of Heidelberg, Im Neuenheimer Feld 130.3, 69120 Heidelberg, Germany

**Keywords:** Endometrial cancer, Amino acids, Acylcarnitine, MiRNA, DNA methylation, Metabolomics, Breast cancer

## Abstract

**Purpose:**

Endometrial carcinoma is the second most common gynecological malignancy. Until today lacking a screening tool. A blood-based biomarker could help address this need.

**Methods:**

The expression levels of 30 acylcarnitines, 18 amino acids, 6 miRNAs, and 7 DNA methylation sites were measured in blood samples from 331 women (20 EC, 14 benign uterine lesions (benign), 140 breast cancers (BC), 157 controls). Areas under the ROC curves (AUC), sensitivity (sens.) and specificity (spec.) were computed to identify the variables best distinguishing.

**Results:**

The best top ten markers for the four comparisons (cancer vs. cancer-free; EC vs. BC, EC vs. controls; EC vs. benign), were identified via AUC. Malonylcarnitine distinguished best patients with EC from controls (AUC: 0.827, sens. 80%, spec. 73.1%) or BC (AUC: 0.819, sens. 84.3%, spec. 80%) being most notable. Tryptophan best differentiated benign from EC (AUC: 0.846, sens. 70%, spec. 92.9%).

**Conclusions:**

The levels of the analyzed blood markers yielded promising results in the detection of EC and warrant further evaluation.

**Supplementary Information:**

The online version contains supplementary material available at 10.1007/s00404-022-06482-8.

## Introduction

Endometrial carcinoma (EC) is the second most common gynecological malignancy among women worldwide, with an increasing prevalence particularly in western countries due to arising obesity [1, 2]. Besides clinical risk factors for EC or endometrial hyperplasia, as precursor lesion [3], such as high BMI, diabetes mellitus, and null parity, cancer predisposition syndromes (CPS), for example Lynch syndrome (LS), are accompanied by an increased life-time risk for EC of up to 62% [4] [5]. In times of panel diagnostics, more healthy family members are identified and confronted with a CPS needing surveillance strategies—especially due to varying 5 year survival from > 90% for low stages versus up to 50% for advanced stages [6]. Screening tools such as transvaginal ultrasound (TVUS) have, so far, failed clinical implementation with a missing survival benefit in the general as well as high-risk populations [7]. TVUS quality standards are particularly limited among obese women, who face a higher overall EC risk and lower EC survival compared to patients of normal weight [8, 9]. EC diagnosis is still mainly guided by the appearance of postmenopausal vaginal bleeding as a clinical symptom and then leading to invasive diagnostics via hysteroscopy and curettage for histological confirmation.

Blood-based biomarkers are a less-invasive, cost-effective, easily retrieved, and repeatable diagnostic approach [10]. Several genomic markers can be measured, such as metabolite levels, miRNA expression, and DNA methylation.

Amino acids are suitable candidates for metabolomic analysis of their essential physiological roles as basic metabolites and regulators [11]. A dysregulation of amino acids has been described previously in the cancer setting. They play an important role not only concerning protein biosynthesis but also as an energy source, maintaining redox balance, epigenetic and immune regulation, and therefore tumor and metastasis growth [12]. Acylcarnitines are the fatty acid esters of carnitine generated by mitochondrial and cytosolic enzymes for the purpose of transporting long-chain fatty acids in the mitochondrial membrane for β-oxidation [13]. Differences in the sera concentration of acylcarnitines were described for patients with obesity and type 2 diabetes mellitus [14]. Since those metabolic alterations are a risk factor for EC, amino acids and acylcarnitines thus seem to be potential parameters for EC.

The human body expresses more than 1000 different miRNAs (miR), a group of non-coding RNAs with a typical length of 23 nucleotides. In plasma, miRs are suspected to be shed from exosomes or tumor cells. MiR expression effects processes such as apoptosis, cell differentiation, and proliferation and therefore cancerogenesis. DNA methylation as a major epigenetic process influences the gene expression of oncogenes and tumor suppressor genes. DNA methylation and miRNA expression have been found to be altered in the tissue and blood of malignancies, including EC patients compared to controls [15–17].

This case-control study evaluates the levels of the aforementioned biomarkers, including amino acids, acylcarnitines, miRNAs, and DNA methylation, to distinguish EC-specific changes compared to breast cancer (BC), benign uterus lesions, and healthy controls. To our knowledge, this is the first study to address the potential of such blood-based biomarkers markers in an EC and BC cohort.

## Material and methods

### Study protocol and population

The study was conducted in the Department of Gynecology and Obstetrics at the University Hospital Heidelberg, Germany, between July 2016 and January 2019. Ethical approval was obtained by the local ethical committees prior to the study start (S-49/2016, S-046/2018, S-684/2018). The study is registered in the German Register for Clinical Studies (DRKS00016964). German-speaking women aged 18 years and older were eligible to participate if written informed consent was provided. Participants were grouped upon diagnosis: EC, BC, benign uterus lesions such as polyps or suspicious endometrium appearance on imaging, or healthy controls without known medical conditions concerning cancer and uterine diseases. Demographic factors and medical anamnesis were assessed using a self-designed questionnaire.

### Blood sample collection and processing

Whole blood samples were collected and processed within 2 h. Serum blood tubes were left to stand for at least 20 min at room temperature for the blood to clot and were then centrifuged at 1300g_max_ for 20 min at 10 °C. The supernatant (one-spin serum) was transferred into 2 ml microcentrifuge tubes followed by a second high-speed centrifugation step at 15,500g_max_ for 10 min at 10 °C to remove cell debris and fragments. The blood clot remaining after initial blood centrifugation was discarded. Following high-speed centrifugation, the cell-free supernatant (double-spin serum) was aliquoted into cryo-vials, snap-frozen in liquid nitrogen, and stored at − 80 °C until use.

### Analyses of amino acids and acylcarnitines

Amino acids and acylcarnitines (Supplement Table I) were determined in plasma by electrospray ionization tandem mass spectrometry (ESI–MS/MS) according to a modified method as previously described [18] using a quattro ultima triple quadrupole mass spectrometer (Micromass, Manchester, UK) equipped with an electrospray ion source and a Micromass MassLynx data system. In brief, 5 µl plasma were placed on a 4.7 mm filter paper punch, dried at room temperature overnight, and extracted with 100 µl of deuterium-labelled standard solution in methanol. After 20 min, the samples were centrifuged and the extract was evaporated to dryness, reconstituted in 60 µl of 3 M HCl in butanol, placed in sealed microtiter plates, and incubated for 15 min at 65 °C. The resulting mixtures were dried, and each residue was finally reconstituted in 100 µl solvent of acetonitrile/water/formic acid (50:50:0.025 v/v/v) prior to measurement.

### DNA isolation and sodium bisulfite conversion

Genomic DNA was isolated from 200 µl whole blood using the Quick-DNA™ miniprep plus kit (Zymo Research, Freiburg, Germany) according to the manufacturer’s recommendation. DNA concentration and quality was measured by NanoDrop ND-100 (peqLab, Erlangen, Germany). 500 ng of genomic DNA were used for the sodium bisulfite conversion using the EZ-96 DNA methylation Gold kit (Zymo Research, Freiburg, Germany) as described by the manufacturer. Bisulfite-converted DNA was amplified by methylation-specific primers (Supplement Table II). Regions between 150 and 300 bp within different genomic regions of MGRN1, RAPSN, RPTOR, FUT7, SLC22A18, S100P and HYAL2 were amplified. A touchdown PCR was used to amplify the regions according to the optimal annealing temperature of the primers. Details of the PCR conditions are available upon request.

### Mass array to measure gene-specific methylation levels

DNA methylation was analyzed by the MassARRAY EpiTYPER assay (Agena BioScience, Hamburg Germany) as previously described [15, 19]. In brief, the PCR products were transcribed *in vitro*, cleaved by RNase A, cleaned by resin, and dispensed to a 384 SpectroCHIP by a nanodispenser. The chips were subjected to matrix-assisted laser desorption ionization time-of-flight mass spectrometry (MALDI-TOF MS). Data were collected by SpectroACQUIRE v3.3.1.3 software and analyzed by the EpiTYPER software.

### MiRNA

MiRNAs were extracted using an Extraction Kit (NucleoSpin miRNA Plasma Machery-Nagel REF 740981.250) following the manufacturer’s protocol. The protocol was complemented by an additional step at the beginning: 1 μl glycogen (10 mg/ml) was added to the aliquot and buffer mix. Qubit measurements were performed using the Qubit Fluorometer (Ref # Q33226) and the Qubit microRNA Assay Kit (Ref # Q32880), both by ThermoFisher Scientific, according to the manufacturer’s protocol. Quantitative measurements using a panel of six miRNAs, miR-148b, miR-200c, miR-320b, miR-375, miR-409, and miR-652, were performed.

### RNA extraction

For measurement of miRNAs, 300 µl of the plasma aliquot was thawed to room temperature. RNA extraction was performed on a safety cabinet using the NucleoSpin^®^ miRNA Plasma Kit from Macherey–Nagel. To the 300 µl sample plasma, 89.5 µl MLP buffer and 0.5 µl glycogen were first added, homogenized, and then incubated for 3 min at room temperature. The further procedure followed the protocol given by the manufacturer. For RT-qPCR, the miRCURY LNA miRNA PCR assay from Qiagen was used in combination with the qPCR-CYBR master mix from Steinbrenner. First, the cDNA was diluted 1:30 by adding 29 µl of RNAse-free water to 1 µl of cDNA. Then, the reaction mix was prepared, which consisted of the following components: 2.5 µl 2 × qPCR-CYBR Master Mix, 0.5 µl PCR Primer Mix, 0.4 µl RNAse-free H2O 0.4, 1.6 µl 1:30 diluted cDNA 1.6. The PCR plate containing the samples was placed in the qTower3 84G.

### Statistical analysis

The objective of the analysis was to determine the variables that perform best—in terms of area under the ROC curve (AUC)—to distinguish between the following groups: cancer patients vs. cancer-free (benign + controls), EC patients vs. BC patients, EC patients vs. patients with benign uterus lesions, and EC patients vs. controls.

AUCs were calculated for all variables and all group comparisons. The 10 variables with the highest AUCs were selected for each comparison. 95% confidence intervals for the AUCs were computed using DeLong’s formula. Additionally, sensitivity and specificity were calculated for the best cut-off value of each variable, which was determined as the value with the highest Youden index.

Baseline characteristics were compared between groups using the analysis of variance for continuous variables and Fisher’s exact test for categorical variables. All analyses were conducted using R version 4.0.4.

## Results

### Patient characteristics

The study cohort comprises 331 women with a mean age of 52 (20–90). Details are shown in Table 1. Mean age differed significantly between groups (*p* < 0.001): 62 for EC vs. 55 for BC, 56 for the patients with benign lesions, and 48 for the healthy controls. BMI distribution showed no significant differences between groups (*p* = 0.06). Descriptively, women with a benign uterine lesion (28 kg/m^2^) or EC (30 kg/m^2^) had a slightly higher BMI than the control group or patients with BC (both 26 kg/m^2^). Diabetic women, who often have a disturbed lipid metabolism disorder and a higher risk of dying from EC than BC [20], were included in the EC, BC and control group without significant differences in the distribution (*p* = 0.42).

**Table 1:** Study collective

Variables	BC	Benign	Control	EC	Total
*N*	140	14	157	20	331
Age (*p* < 0.001)					
* N*	140	14	157	20	331
Mean ± SD	55 ± 14	56 ± 12	48 ± 14	62 ± 9	52 ± 14
Range	28–90	35–77	20–84	50–80	20–90
BMI (*p* = 0.06)					
* N*	140	14	157	20	331
Mean ± SD	26 ± 5.3	28 ± 7.6	26 ± 7.1	30 ± 6.2	26 ± 6.4
Range	17–44	19–44	16–69	21–42	16–69
Diabetes (*p* = 0.42)					
No (%)	121 (86)	14 (100)	131 (83)	17 (85)	283 (85)
Yes (%)	19 (14)	0 (0)	26 (17)	3 (15)	48 (15)

*BC* Breast cancer, *Benign* Women with benign uterine lesions, *EC* Endometrial cancer, *N* Number, *SD* Standard deviation, *BMI* Body mass index

### Analyses of the measurements of amino acids, acylcarnitines, miRNA and DNA methylation

**Fig. 1:**
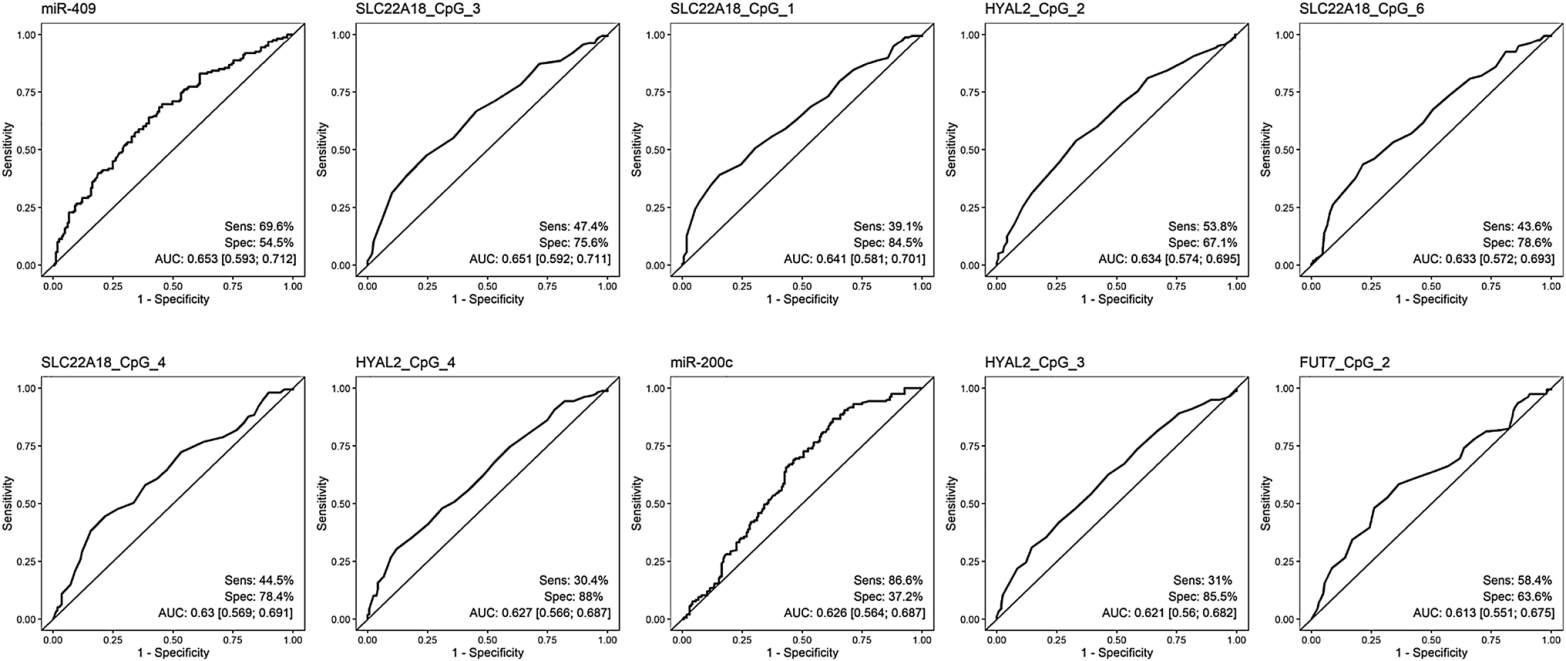
ROC curves for cancer patients vs. cancer-free. *Sens* Sensitivity, *Spec* specificity, *AUC* Area under the curve

General cancer-related changes were recognized via a pooled analysis of all cancer patients (EC and BC) compared to cancer-free women (healthy controls and women with benign uterus lesions). The top ten from the univariable analyses for the cancer versus cancer-free comparison composed of two miRNAs (409 and 200c) and 8 CpG sites (4 CpGs at SLC22A18, 3 at HYAL2, and 1 at FUT7). The ROC curves together with the AUCs and their 95% confidence intervals as well as the sensitivities and specificities for the best cut-off values are shown in Fig. 1. The AUCs for the ten best variables reached a maximum of 0.653 for miRNA-409, with a sensitivity of 69.6% and specificity of 54.4%.

**Fig. 2:**
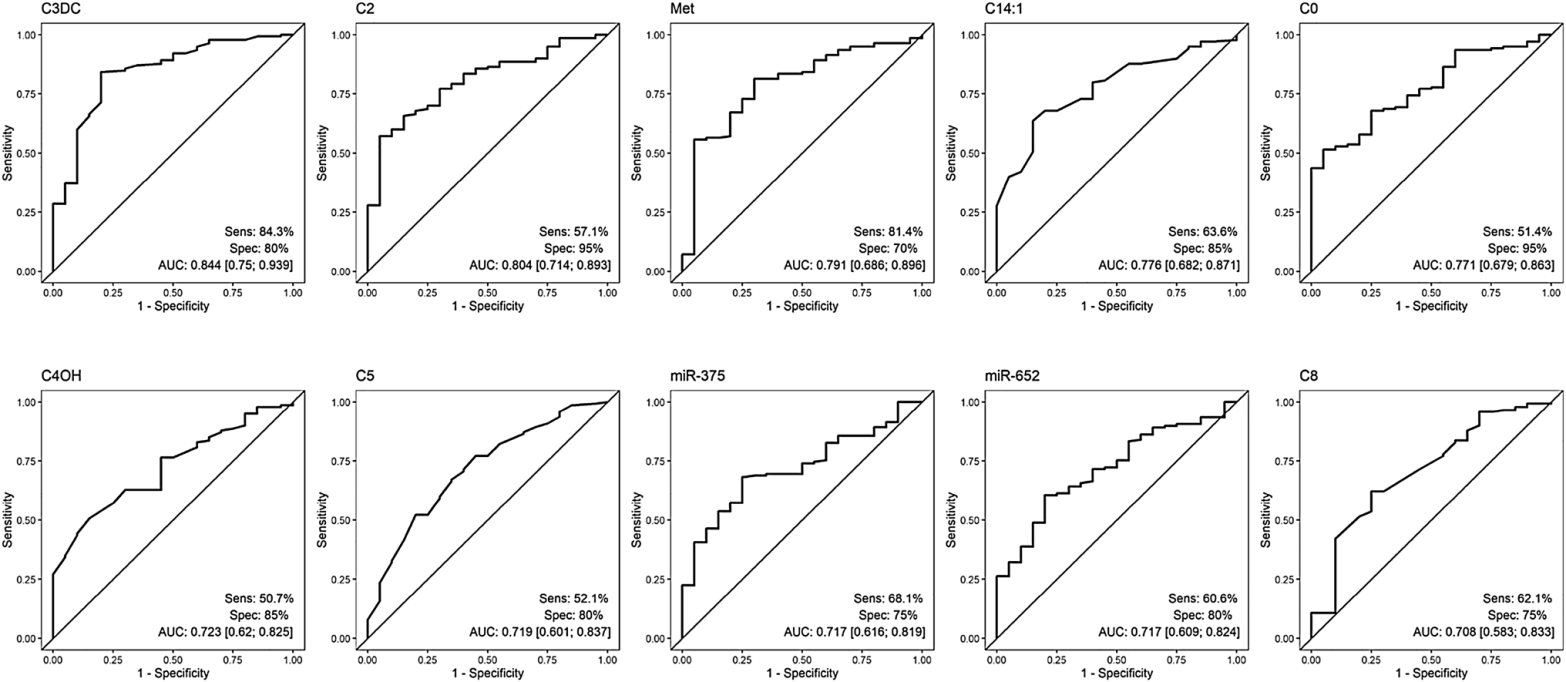
ROC classifier curves of the analyses of EC vs BC. *EC* Endometrial Cancer, *BC* Breast Cancer, *Sens* Sensitivity, *Spec* specificity, *AUC* Area under the curve, *C3DC* Malonylcarnitine, *C2* Acetylcarnitine, *Met* Methionine, *C14:1* Tetradecenoylcarnitine, *C0* Carnitine, *C4OH* 3-OH-Butylrylcarnitine, *C5* Isovalerycarnitine, *C8* Octanylcarnitine

To determine which differences were EC-specific and not generally attributable to cancer, we compared EC with BC patients. The ten best variables distinguishing between EC and BC patients were: malonylcarnitin, acetylcarnitin, methionine, tetradecenoylcarnitin, carnitine, 3-OH-butylrylcarnitine, isovalerycarnitine, miR-375, miR-652, and octanylcarnitine, with AUCs between 0.708 and 0.844 (Fig. 2). The amino acid methionine was the only variable in the top ten that was higher in BC patients. All other top ten parameters (malonylcarnitin, acetylcarnitin, tetradecenoylcarnitin, carnitine, 3-OH-butylrylcarnitine, isovalerycarnitine, miR-375, miR-652 and octanylcarnitine) were higher among the EC group (Supplement Table III). Malonylcarnitin had the best AUC and achieved a sensitivity of 84.3% and a specificity of 80%.

**Fig. 3:**
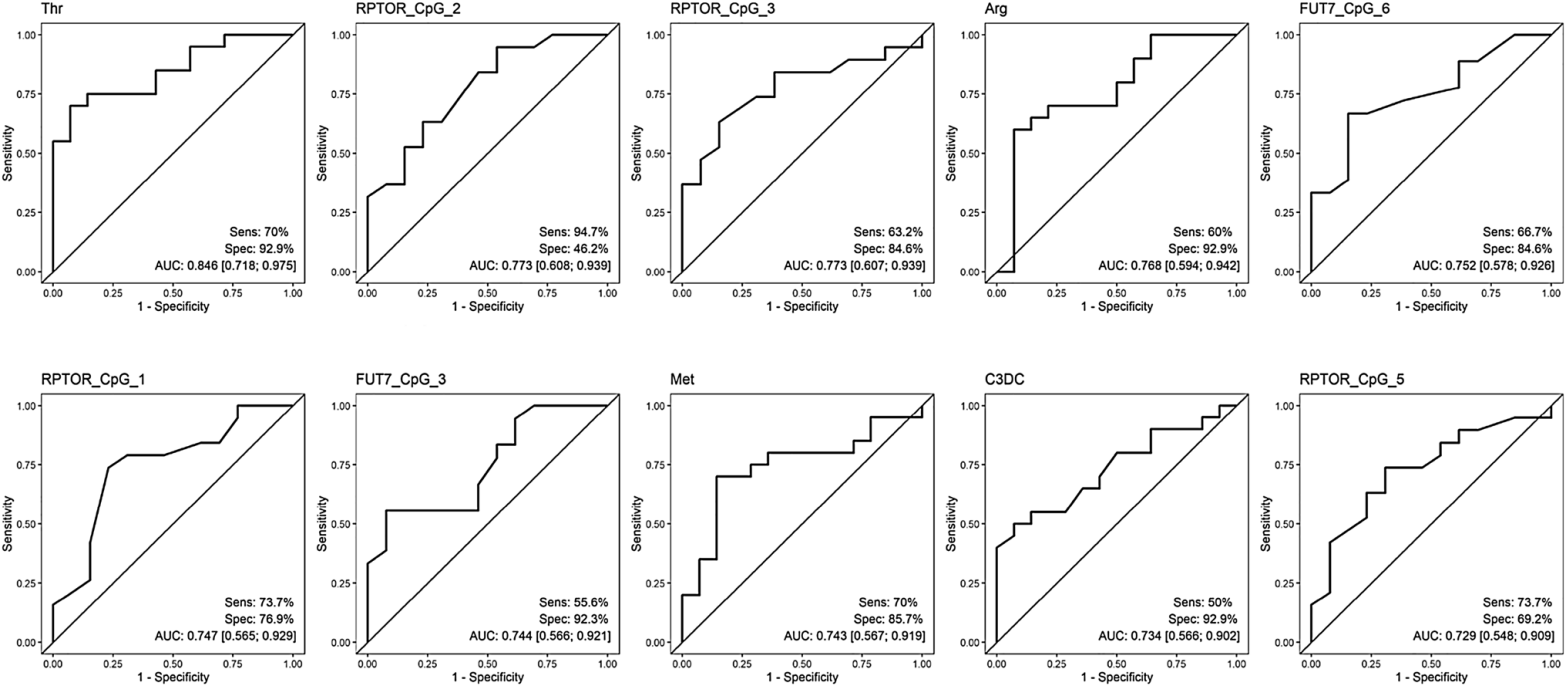
ROC classifier curves of the analyses of EC vs benign. *EC* Endometrial Cancer, *Benign* Women with benign uterine lesions, *Sens* Sensitivity, *Spec* specificity, *AUC* Area under the curve, *Thr* Tryptophan, *Arg* Arginine, *Met* Methionine, *C3DC* Malonylcarnitine

The clinical need to differentiate malignant from benign sonographic findings was addressed by the serum analysis of EC patients compared to women with benign uterus lesions. The ten best variables reached AUCs between 0.729 and 0.846 (Fig. 3). By far, tryptophan had the best AUC, the associated sensitivity was 70%, and the specificity was 92.9%. Tryptophan, arginine, and methionine were elevated among women with benign uterine lesions compared to EC patients (Supplement Table IV). DNA methylation of four CpG sites at RPTOR, two CpG sites at FUT7 and malonylcarnitine, were higher among EC patients.

**Fig. 4:**
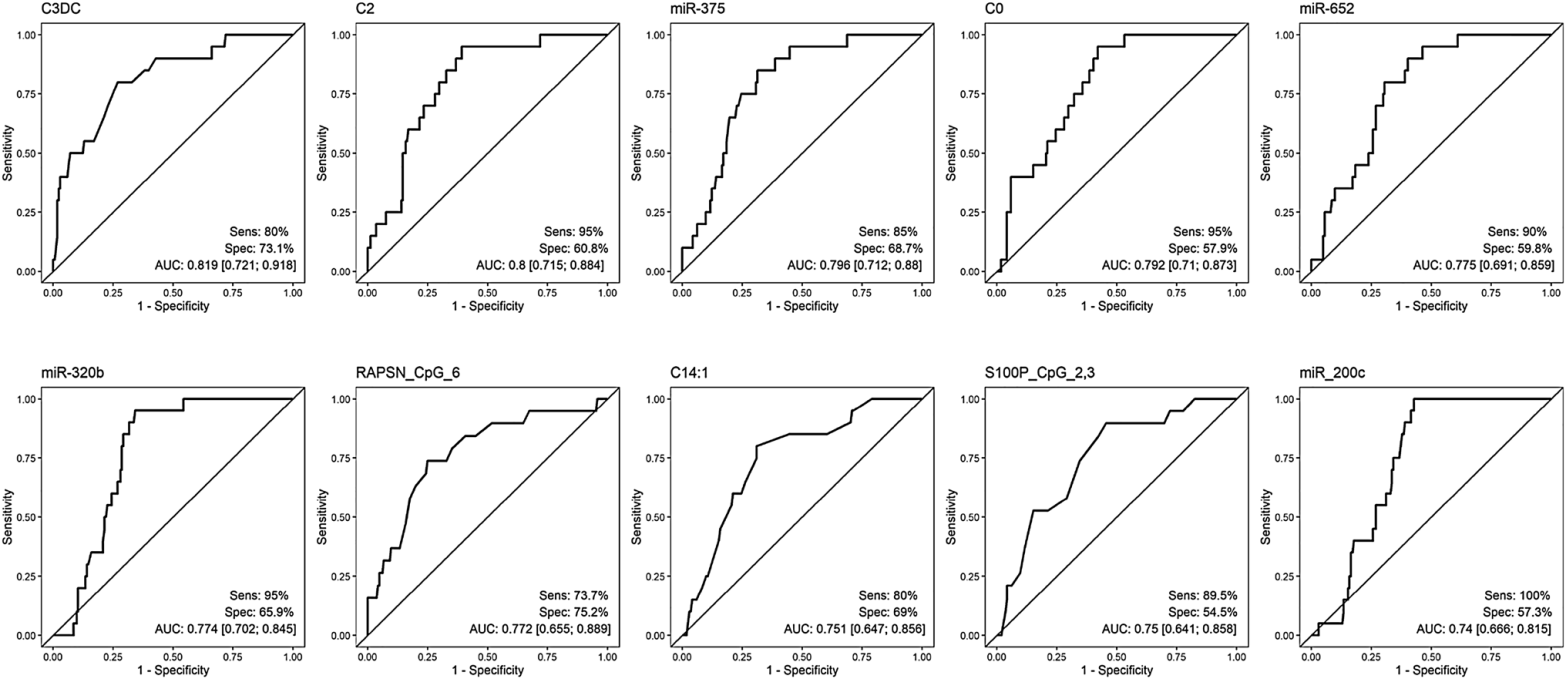
ROC classifier curves of the analyses of EC vs controls. *EC* Endometrial Cancer, *Sens* Sensitivity, *Spec* specificity, *AUC* Area under the curve, *C3DC* Malonylcarnitine, *C2* Acetylcarnitine, *C0* Carnitine, *C14:1* Tetradecenoylcarnitine

To identify potential blood-based EC screening parameters, we compared EC patients with healthy controls (Fig. 4). The ten best variables were: malonylcarnitine, miR-375, acetylcarnitine, carnitine, miR-652, miR-320b, one CpG site at RAPSN and one at S100P, miR-200c and tetradecenoylcarnitine, with AUCs between 0.74 and 0.819. Again, malonylcarnitine had the highest AUC, with corresponding sensitivity of 80% and specificity of 73.1%.

### Amino acids were lower in EC patients compared to BC and the benign group

Interestingly, all amino acids appearing in the top ten were lower in EC patients than in the corresponding comparison group. The low value of the amino acids methionine, arginine, and tryptophan among EC patients compared to BC and women with benign uterus lesions identified (Supplement Table III + IV) might be explained by the hypermetabolic activity of EC cells and their increased gluconeogenesis and protein catabolism [21].

### Acylcarnitines were elevated in the blood of EC patients

In all three comparisons with EC (vs BC, vs benign, vs controls), acylcarnitines were higher in the EC group than in the respective comparison group. An alteration in the concentration of acylcarnitines in the blood of women with an EC has already been shown in some studies [22]. They play an important role in cancerogenesis by altering beta-oxidation, energy expenditure, and lipolysis. The fact that all the carnitines were higher in EC than in BC emphasizes the importance of the fatty acid metabolism in EC but not in cancer in general (Supplement Table III).

### Altered DNA methylation in EC patients

DNA methylation plays a key role in gene expression regulation by modulating the chromatin structure and influencing functional changes in endometrial tissue. We found that SLC22A18, encoding an orphan transporter, and HYLA, serving as an oncogene involved in cell mobility and cancer progression through chemokinesis causing angiogenesis and metastasis [23], were hypomethylated in cancer patients in general compared to cancer-free women (Supplement Table V). The receptor-associated protein of the synapse (RAPSN) gene was hypomethylated in EC patients compared to the controls and BC patients (Supplement Table III + VI). Hypomethylation of RAPSN has been previously described for BC compared to healthy controls [24]. The RPTOR (Regulatory Associated Protein Of MTOR Complex) gene encodes for a protein regulating cell growth as well as apoptosis depending on the nutrient and insulin [25]. FUT7 plays a major role in the migration of tumor cells from blood vessels to the surrounding tissue and was conclusively hypermethylated among our EC patients compared to women with benign changes (Supplement Table V). Changes in RPTOR and FUT7 methylation have not been previously described for EC. S100P encodes for the S100 Calcium Binding Protein P through the binding of Ca ions. It mediates invasion and thereby tumor and metastasis growth [26]; hypomethylation was detected for EC compared to the control group (Supplement Table VI).

### MiRNA concentration differ in cancer patients compared to controls

Dysregulated miRNAs are said to be causally related to the pathogenicity of many kinds of cancer. MiR-200c is important for the transition from epithelial-to-mesenchymal and vice versa [27]. In our study, it was lower in cancer patients compared to controls (Supplement Table V + VI). The same results were detected for miR-409, supporting its described effect as a tumor suppressor in ovarian cancer (Supplement Table V)[28]. The miR-375 gene is important for insulin secretion and glycogen synthase [29] and acts as a tumor suppressor by downregulating numerous oncogenes, e.g. PDK1, JAK2, IGF1R, AEG-1, and suppressing the PI3K/Akt pathway [30]. It was higher in EC than in BC patients or controls (Supplement Table III + VI). Another elevation in EC compared to controls was found for miR-652, which is known to promote tumor and metastasis growth via retinoic acid receptor-related orphan receptor A and for miR-320b (Supplement Table VI).

## Discussion

Early cancer diagnosis of endometrium cancer (EC) remains a major challenge and may result in a more favorable disease outcome. Therefore, novel and minimally invasive techniques such as blood-based biomarkers measuring circulating amino acids, acylcarnitines, miRNA, and DNA methylation are potential candidates to broaden the spectrum of diagnostic approaches. Here, expression profiles specific for malignant and benign endometrium lesions as well as for breast cancer (BC) were detected.

First, patterns in women with cancer (EC + BC) were observed compared to patterns of cancer-free women to identify cancer patterns in general. Encouragingly, none of the top ten parameters of this comparison reappeared in any other analysis. However, with low AUCs of 0.613–0.653 and a sensitivity of 69.6% and specificity of 54.4% for the best performing marker, miR-409, our results are inferior to other publications on large scale multivariable analyses on other tumor types such as CancerSEEK, with AUCs ranged from 69 to 98% [31]. It should be noted, however, that EC was not included in their liquid biopsy approach, and tumor stage is an important factor impacting sensitivity, which was not taken into account in our rather small cohort.

In our second analysis, we compared patterns of women with EC to BC, reaching AUCs of up to 0.844 and a sensitivity of 84.3% and specificity of 80% (malonylcarnitin) with a potential for further evaluations in cohorts at high risk for cancer. In our third analysis of women with EC compared to those with a benign uterus lesion, tryptophan, the best performing marker, almost reaches the values of a transvaginal ultrasound examination (tryptophan sensitivity of 70% and a specificity of 92.2% versus 80.5% and 86.2% respectively a large study in the UK) [32]. These might open options and warrant further evaluation, especially in a cohort with transvaginal sonography limitations, e.g. obese women. Furthermore, it may help to distinguish benign from malignant uterus lesions without hysteroscopy, which is particularly helpful for women for whom an operation would be very risky due to conditions such as obesity or other comorbidities. We hypothesize that a through combination of tryptophan with sonography, one could certainly substantially improve the diagnostic power.

To date, there is no established screening tool for EC. The most widely used marker is HE4, for which high preoperative levels of HE4 are associated with advanced clinical stage and worse overall and recurrence-free survival [33], AUCs of 0.76–0.97 (sensitivity 46–91%; specificity 66–100%) in screening context were described [34]. Furthermore. With an AUC of 0.83 and a sensitivity of 80% and specificity of 73.1% for malonylcarnitine to distinguish between healthy and EC patients in our study, this marker compares well with HE4 and could probably be considerably improved in specificity and sensitivity by combining it with HE4.

Our blood-based findings confirmed the current knowledge on tryptophan, arginine, and methionine and their downregulation among EC patients. The depletion of tryptophan is argued to suppress antitumor immune responses through the accumulation of immunosuppressive catabolites causing anergy and apoptosis in T-cells [35, 36]. *In vitro*, the downregulation of arginine caused increased cell motility and invasion capability, easing the development of EC [37]. The potential of methionine as a blood-based EC-specific marker and its use to differentiate EC from BC when positive for cancer in a pan-cancer panel needs further investigation.

Acylcarnitines are crucial signaling molecules in the energy and fatty acid metabolism and transport, and all were higher in the pattern of our EC group compared to BC, controls and women with benign uterus lesions. Malonylcarnitine is a precursor of Malonyl‐CoA that causes malonylation of mTOR by its increase, and thus has a positive effect on the angiogenesis and the pathogenesis of cancer [38]. Free carnitine was described to have a protective role in BC [39]. This does not seem to be the case for EC, and it is considered cancerogenic as described for colorectal cancer [40], possibly due to underlying molecular pathologic patterns. Arioz et al. found carnitine to be lower in the blood of EC patients compared to controls depending on the FIGO state [41]. They suspected that carnitine depletion leads to an accumulation of toxic metabolites and a disruption of the DNA repair system. This contrasts with our results. However, their distribution of diabetics was unequal and smaller in the EC group, causing a selection bias, as it is known that diabetes is associated with low carnitine blood levels [42].

Age is a risk factor for EC and associated with lower OS at EC patients [43], and tetradecenoylcarnitine in the plasma was found to be correlated with age [44], which fits with our results considering that EC patients were older than all other groups. Furthermore, it is elevated with impaired glucose tolerance [45], a precursor of diabetes melitus, which in turn is a risk factor for the development of EC. BC patients in our cohort were younger and had lower tetradecenoylcarnitine than EC, which is concordant with previous case–control studies suggesting increased β-oxidation for energy production at EC [46]. Butyrylcarnitine is elevated in metastatic ovarian cancer [47] and associated with a high BMI [48], supporting our results insofar that the EC patients had a higher BMI than the BC and higher values of 3-hydorxy-butyrylcarnitine. In contrast to our results, one study found lower levels of isovalerylcarnitine in the serum of EC patients compared to controls without BMI significance [49]. Interestingly, a positive predictive value of 100% was described by Park et al. for lower octanoylcarnitine and BC diagnose, which was attributed to high consumption by BC offering metabolic flexibility [50] independent from glucose. This agrees with our detected decrease among controls compared to BC patients. To date, there are no studies describing a correlation between EC and octanoylcarnitine.

Aberrant DNA methylation changes at cytosines result in deregulation of important apoptotic proteins during endometrial carcinogenesis and thus apoptosis resistance. Similar to other carcinoma, in EC various tumor suppressor gene promotors (MLH1, PTEN, p16, APC, MGMT, RASSF1, PR and CDH1) are hypermethylated, whereas oncogene promotors (BMP, CTCFL, PARP1, CASP8) are hypo- or demethylated [51]. SLC22A18 has an inhibiting effect on colony formation and causes a G2/M breakpoint, and it is also associated with a poor prognosis when downregulated in BC patients [52]. According to our study, HYLA2 seems to act as an oncogene, as described before, with a hypomethylation in cancer patients [53]. The RPTOR gene encodes for a protein regulating cell growth and also apoptosis depending on the nutrient and insulin [25]. Differences in the methylation of RPTOR have not been described for EC. The apoptosis triggered by RPTOR hyperactivation could thus play a role in the development of EC [54]. There are no previous findings for FUT7 and EC. However, the hypermethylation in EC patients goes along with its important known role in cell cycle progression in HCC [55]. The hypomethylation pattern of RAPSN in EC has been previously described for BC and lung cancer, yet its mechanism is not fully understood. It is likely that CDK5 and adenylate cyclase 9 are part of the link between RAPSN and tumorigenesis via N-glycosylation [56]. To our knowledge, this is the first study describing RAPSN and S100P methylation differences in patients with EC.

A recent review by Hutt et al.[57] described numerous miRNAs with diagnostic potential for EC [58, 59] and for circular RNAs are said to provide new insights for EC treatment [60]. The decreased miR-200c blood levels in our cancer patients were described for hepatic cellular cancer tissue, and its potential as a prognostic marker or even as a therapeutic target discussed [61]. The findings of lower miR-409 are concordant to previous results in EC tissue and BC cell lines compared to healthy tissue [62] [63]. MiR-375 was higher among EC patients compared to the BC and the control group. In BC patients it is found to be elevated compared to controls and correlated positively with the estrogen, progesterone, and Her2 receptor expression of the carcinoma and obesity [64]. Since it has been shown in BC that miR-375 increase is correlated with obesity, this could be an explanation for the increase among EC patients in our study, as they tended to be more obese than the other groups. The elevation of miR-652 in the blood tissue of EC patients and its association with shorter overall and progression-free survival was previously described by Sun et al. [65], supporting our results. The age-dependent elevation of miR-320b in the blood of older BC patients [66] fits with our results as EC patients are older than the control group.

In conclusion, the analyses of acylcarnitines, amino acids, miRNA, and DNA methylation yield promising results in our current study. They warrant further evaluation in a larger, prospective, multicentric setting with standardized sample collection and age and BMI matched groups to evaluate their clinical potential, especially in the situation where current diagnostic tools fail clinical applicability. Furthermore, with a larger study collective multivariable analysis can be performed to further address sensitivity and specificity. Blood-based approaches could be beneficial not only for a high-risk population predisposed to EC but also for obese patients for whom the current diagnostic standards have limited informative value and a higher risk of intervention.

## Supplementary Information

Below is the link to the electronic supplementary material.

Supplementary file1 (DOCX 37 KB)

## References

[1] Siegel RL, Miller KD, Jemal A (2015). Cancer statistics, 2015. CA Cancer J Clin.

[2] Bray F (2018). Global cancer statistics 2018: GLOBOCAN estimates of incidence and mortality worldwide for 36 cancers in 185 countries. CA Cancer J Clin.

[3] Nees LK (2022). Endometrial hyperplasia as a risk factor of endometrial cancer. Arch Gynecol Obstet.

[4] Raglan O (2019). Risk factors for endometrial cancer: an umbrella review of the literature. Int J Cancer.

[5] Aarnio M (1999). Cancer risk in mutation carriers of DNA-mismatch-repair genes. Int J Cancer.

[6] Dowdy SC (2014). Improving oncologic outcomes for women with endometrial cancer: realigning our sights. Gynecol Oncol.

[7] Helder-Woolderink JM (2013). The additional value of endometrial sampling in the early detection of endometrial cancer in women with lynch syndrome. Gynecol Oncol.

[8] Secord AA (2016). Body mass index and mortality in endometrial cancer: a systematic review and meta-analysis. Gynecol Oncol.

[9] Nattenmüller J (2018). Visceral abdominal fat measured by computer tomography as a prognostic factor for gynecological malignancies?. Oncotarget.

[10] Zhang A (2012). Modern analytical techniques in metabolomics analysis. Analyst.

[11] Miyagi Y (2011). Plasma free amino acid profiling of five types of cancer patients and its application for early detection. PLoS ONE.

[12] Lieu EL (2020). Amino acids in cancer. Exp Mol Med.

[13] Wilcken B (2003). Screening newborns for inborn errors of metabolism by tandem mass spectrometry. N Engl J Med.

[14] Mihalik SJ (2010). Increased levels of plasma acylcarnitines in obesity and type 2 diabetes and identification of a marker of glucolipotoxicity. Obesity (Silver Spring).

[15] Ehrich M (2005). Quantitative high-throughput analysis of DNA methylation patterns by base-specific cleavage and mass spectrometry. Proc Natl Acad Sci USA.

[16] Roth C (2010). Circulating microRNAs as blood-based markers for patients with primary and metastatic breast cancer. Breast Cancer Res.

[17] Wilczynski M (2018). Association of microRNA-200c expression levels with clinicopathological factors and prognosis in endometrioid endometrial cancer. Acta Obstet Gynecol Scand.

[18] Sauer SW (2006). Intracerebral accumulation of glutaric and 3-hydroxyglutaric acids secondary to limited flux across the blood-brain barrier constitute a biochemical risk factor for neurodegeneration in glutaryl-CoA dehydrogenase deficiency. J Neurochem.

[19] Wilhelm T (2016). Epigenetic silencing of AKAP12 in juvenile myelomonocytic leukemia. Epigenetics.

[20] Gallagher EJ, LeRoith D (2015). Obesity and Diabetes: the increased risk of cancer and cancer-related mortality. Physiol Rev.

[21] Aoyagi T (2015). Cancer cachexia, mechanism and treatment. World J Gastrointest Oncol.

[22] Raffone A (2020). Metabolomics in endometrial cancer diagnosis: a systematic review. Acta Obstet Gynecol Scand.

[23] Chowdhury B (2016). Hyaluronidase 2 (HYAL2) is expressed in endothelial cells, as well as some specialized epithelial cells, and is required for normal hyaluronan catabolism. Histochem Cell Biol.

[24] Tang Q (2016). DNA methylation array analysis identifies breast cancer associated RPTOR, MGRN1 and RAPSN hypomethylation in peripheral blood DNA. Oncotarget.

[25] Stelzer G (2016). The genecards suite: from gene data mining to disease genome sequence analyses. Curr Protoc Bioinformatics.

[26] Jiang H (2012). Calcium-binding protein S100P and cancer: mechanisms and clinical relevance. J Cancer Res Clin Oncol.

[27] Liu W (2018). Correlation between miR-200 family overexpression and cancer prognosis. Dis Markers.

[28] Cheng Y (2018). MiRNA-409-3p enhances cisplatin-sensitivity of ovarian cancer cells by blocking the autophagy mediated by Fip200. Oncol Res.

[29] Baroukh NN, Van Obberghen E (2009). Function of microRNA-375 and microRNA-124a in pancreas and brain. Febs j.

[30] Canlorbe G (2016). Identification of microRNA expression profile related to lymph node status in women with early-stage grade 1–2 endometrial cancer. Mod Pathol.

[31] Cohen JD (2018). Detection and localization of surgically resectable cancers with a multi-analyte blood test. Science.

[32] Jacobs I (2011). Sensitivity of transvaginal ultrasound screening for endometrial cancer in postmenopausal women: a case-control study within the UKCTOCS cohort. Lancet Oncol.

[33] Insin P, Yimyam Y, Prueksaritanond N (2021). Association of preoperative serum HE4 levels on the survival of patients with endometrial cancer. Arch Gynecol Obstet.

[34] Degez M (2021). Endometrial cancer: a systematic review of HE4. REM and REM-B Clinica Chimica Acta.

[35] Platten M, Wick W, Van den Eynde BJ (2012). Tryptophan catabolism in cancer: beyond IDO and tryptophan depletion. Cancer Res.

[36] de Jong RA (2011). Serum tryptophan and kynurenine concentrations as parameters for indoleamine 2,3-dioxygenase activity in patients with endometrial, ovarian, and vulvar cancer. Int J Gynecol Cancer.

[37] Ohshima K (2017). Argininosuccinate synthase 1-deficiency enhances the cell sensitivity to arginine through decreased deptor expression in endometrial cancer. Sci Rep.

[38] Bruning U (2018). Impairment of angiogenesis by fatty acid synthase inhibition involves mTOR malonylation. Cell Metab.

[39] Erbas H (2007). Protective role of carnitine in breast cancer via decreasing arginase activity and increasing nitric oxide. Cell Biol Int.

[40] Amir Hashim NA (2021). Global metabolomics profiling of colorectal cancer in Malaysian patients. Bioimpacts.

[41] Arioz DT (2015). L-Carnitine: a new insight into the pathogenesis of endometrial cancer. Arch Gynecol Obstet.

[42] Steiber A, Kerner J, Hoppel CL (2004). Carnitine: a nutritional, biosynthetic, and functional perspective. Mol Aspects Med.

[43] Hein A (2020). Risk of postmenopausal hormone therapy and patient history factors for the survival rate in women with endometrial carcinoma. Arch Gynecol Obstet.

[44] Liu W (2018). Metabolic biomarkers of aging and aging-related diseases in Chinese middle-aged and elderly men. J Nutr Health Aging.

[45] Mai M (2013). serum levels of acylcarnitines are altered in prediabetic conditions. PLoS ONE.

[46] Cala MP (2018). Multiplatform plasma metabolic and lipid fingerprinting of breast cancer: a pilot control-case study in Colombian Hispanic women. PLoS ONE.

[47] Fong MY, McDunn J, Kakar SS (2011). Identification of metabolites in the normal ovary and their transformation in primary and metastatic ovarian cancer. PLoS ONE.

[48] Moore SC (2014). Human metabolic correlates of body mass index. Metabolomics.

[49] Gaudet MM (2012). Analysis of serum metabolic profiles in women with endometrial cancer and controls in a population-based case-control study. J Clin Endocrinol Metab.

[50] Park J (2019). Plasma metabolites as possible biomarkers for diagnosis of breast cancer. PLoS ONE.

[51] Caplakova V (2016). DNA methylation machinery in the endometrium and endometrial cancer. Anticancer Res.

[52] He H (2011). Low expression of SLC22A18 predicts poor survival outcome in patients with breast cancer after surgery. Cancer Epidemiol.

[53] Schott S (2017). HYAL2 methylation in peripheral blood as a potential marker for the detection of pancreatic cancer: a case control study. Oncotarget.

[54] Thedieck K (2013). Inhibition of mTORC1 by astrin and stress granules prevents apoptosis in cancer cells. Cell.

[55] Li D (2018). α-1,3-Fucosyltransferase-VII siRNA inhibits the expression of SLex and hepatocarcinoma cell proliferation. Int J Mol Med.

[56] Qiao R (2020). The association between RAPSN methylation in peripheral blood and early stage lung cancer detected in case-control cohort. Cancer Manage and Res.

[57] Hutt S (2019). The role of biomarkers in endometrial cancer and hyperplasia: a literature review. Acta Oncol.

[58] He Z (2017). MiR-944 acts as a prognostic marker and promotes the tumor progression in endometrial cancer. Biomed Pharmacother.

[59] Lu L (2016). Oncogenic function of miR-301 to predicts poor prognosis of endometrial cancer. Int J Clin Exp Med.

[60] Wei D (2021). Circular RNA circ_0000043 promotes endometrial carcinoma progression by regulating miR-1271-5p/CTNND1 axis. Arch Gynecol Obstet.

[61] Li Y, Bai W, Zhang J (2017). MiR-200c-5p suppresses proliferation and metastasis of human hepatocellular carcinoma (HCC) via suppressing MAD2L1. Biomed Pharmacother.

[62] Zhang C, Wang B, Wu L (2019). MicroRNA-409 may function as a tumor suppressor in endometrial carcinoma cells by targeting Smad2. Mol Med Rep.

[63] Yu H (2017). MicroRNA-409-5p is upregulated in breast cancer and its downregulation inhibits cancer development through downstream target of RSU1. Tumor Biology.

[64] Ali OS (2018). MicroRNAs 182 and 375 sera expression as prognostic biochemical markers in breast cancer. Clin Breast Cancer.

[65] Sun X (2018). MiR-652 promotes tumor proliferation and metastasis by targeting RORA in endometrial cancer. Mol Cancer Res.

[66] Hatse S (2014). Circulating microRNAs as easy-to-measure aging biomarkers in older breast cancer patients: correlation with chronological age but not with fitness/frailty status. PLoS ONE.

