# Supplementary material for: Potential of blood-based biomarker approaches in endometrium and breast cancer: a case-control comparison study
Source: Arch Gynecol Obstet. 2022 Mar 13;306(5):1623–32. doi: 10.1007/s00404-022-06482-8 (PMC9519681; doi:10.1007/s00404-022-06482-8)
Supplement: Supplementary file 1 — Supplementary file1 (DOCX 37 KB) [file 404_2022_6482_MOESM1_ESM.docx]

## Supplements

**Table I List of the analysed amino acids and acylcarnitines**

| **Acylcarnitines** | **Amino acids** |
| --- | --- |
| carnitine | glycine |
| acetylcarnitin | ornithine |
| propionylcarnitine | succinylaceton |
| malonylcarnitine | arginine |
| butyrylcarnitine /isobutyrylcarnitine | citrulline |
| 3-OH-butylrylcarnitine | homocitrulline |
| tiglylcarnitine | alanine |
| isovalerycarnitine | proline |
| 2-OH-3-methyl-butyrylcarnitine | valine |
| hexanoylcarnitine | threonine |
| octenoylcarnitine | leucine/isoleucine |
| decenoylcarnitine | methionine |
| decanoylcarnitine | histidine |
| methylglutarylcarnitine | phenylalanine |
| dodecanoylcarnitine | tyrosine |
| tetradecenoylcarnitine | aspartate |
| tetradecanoylcarnitine | tryptophan |
| hydroxytetradecanoylcarnitine |  |
| hexadecenoylcarnitine |  |
| hexadecanoylcarnitine |  |
| hydroxyhexadecenoylcarnitine |  |
| hydroxyhexadecanoylcarnitine |  |
| octadecenoylcarnitine |  |
| octadecanoylcarnitine |  |
| hydroxyoctadecenoylcarnitine |  |
| hydroxyoctadecanoylcarnitine |  |
| octadecadienylcarnitine |  |
| 3-hydroximethylglutarate |  |

**Table II: Bisulfite specific primers**

|  | Primers | PrimerID | Sequences |
| --- | --- | --- | --- |
| **S100P** | sense | S100P_F | aggaagagagGGAAGGTGGGTTTGAATTTAGTATT |
|  | antisense | S100P_R | cagtaatacgactcactatagggagaaggctCTATCCCTCTTACCTCTAAACCCCT |
| **SLC22A18** | sense | SLC22A18_F | aggaagagagTAAGTGGAATTTTGGTATTTTTGGA |
|  | antisense | SLC22A18_R | cagtaatacgactcactatagggagaaggctCACTCCAAACCTAAACTCACCTCTA |
| **FUT7** | sense | FUT7_F | aggaagagagGAAGAGGAAGGGATTTAGTTTGAAG |
|  | antisense | FUT7_R | cagtaatacgactcactatagggagaaggctACAAACCTTAACCTCCCAAAATACT |
| **RPTOR** | sense | RPTOR_F | aggaagagagGTGGGGTTTTTGTAGTAGTTGAGA |
|  | antisense | RPTOR_R | cagtaatacgactcactatagggagaaggctTAATAACCCAAAACCAAACCCTAAC |
| **MGRN1** | sense | MGRN1_F | aggaagagagTTTTGGGGTATAAGGGAAGTTTAAG |
|  | antisense | MGRN1_R | cagtaatacgactcactatagggagaaggctCCTAACCAACAAAAAACCTAAAAAA |
| **RAPSN** | sense | RAPSN_F | aggaagagagGATTTTTAGTTGGTGAGAGGTTTGA |
|  | antisense | RAPSN_R | cagtaatacgactcactatagggagaaggctAAAACCACTAAATTACCCAACCAAA |
| **HYAL2** | sense | HYAL2_F | aggaagagagTTTTAAATTTAGTAGGGTGTGAGAGGA |
|  | antisense | HYAL2_R | cagtaatacgactcactatagggagaaggctCTCATCCATATTATAAAAAACCCCC |

**Table III: Descriptive statistic EC vs BC. N=Number, SD=Standard Deviation, MV=missing values, C3DC=Malonylcarnitine, C2=Acetylcarnitine, Met=Methionine, C14:1=Tetradecenoylcarnitine, C0=Carnitine, C4OH=3-OH-Butylrylcarnitine, C5=Isovalerycarnitine, C8=Octanylcarnitine**

| **Variables** | **EC** | **BC** |
| --- | --- | --- |
| **N** | 20 | 140 |
| C3DC |  |  |
| mean ± sd [Range] | 0.26 ± 0.084 [0.12-0.41] | 0.16 ± 0.068 [0 0.4] |
| mv | 0 | 0 |
|  |  |  |
| C2 |  |  |
| mean ± sd [Range] | 9.3 ± 3.1 [4.6-17] | 6.4 ± 2.4 [2.6-16] |
| mv | 0 | 0 |
| Met |  |  |
| mean ± sd [Range] | 14 ± 3.5 [8.5-25] | 18 ± 5.1 [7.3-46] |
| mv | 0 | 0 |
| C14:1 |  |  |
| mean ± sd [Range] | 0.11 ± 0.035 [0.05-0.17] | 0.073 ± 0.038 [0.02-0.23] |
| mv | 0 | 0 |
| C0 |  |  |
| mean ± sd [Range] | 32 ± 6.8 [22-48] | 25 ± 7 [12-43] |
| mv | 0 | 0 |
| C4OH |  |  |
| mean ± sd [Range] | 0.16 ± 0.079 [0.07-0.35] | 0.11 ± 0.07 [0-0.4] |
| mv | 0 | 0 |
| C5 |  |  |
| mean ± sd [Range] | 0.15 ± 0.05 [0.06-.024] | 0.11 ± 0.044 [0.03-0.24] |
| mv | 0 | 0 |
| miR_375 |  |  |
| mean ± sd [Range] | 32 ± 1.7 [28-36] | 30 ± 3.2 [22-36] |
| mv | 0 | 2 |
| miR_652 |  |  |
| mean ± sd [Range] | 31 ± 1.9 [27-36] | 29 ± 3 [22-35] |
| mv | 0 | 3 |
| C8 |  |  |
| mean ± sd [Range] | 0.17 ± 0.072 [0.075-0.33] | 0.13 ± 0.051 [0.05-0.37] |
| mv | 0 | 0 |

**Table IV: Descriptive statistic EC vs benign. N=Number, SD=Standard Deviation, MV=missing values, Thr= Tryptophan, Arg=Arginine, Met=Methionine, C3DC= Malonylcarnitine**

| **Variables** | **Benign** | **EC** |
| --- | --- | --- |
| N | 14 | 20 |
| Thr |  |  |
| mean ± sd [Range] | 159 ± 52 [112-277] | 113 ± 27 [77-188] |
| mv | 0 | 0 |
| RPTOR_CpG_2 |  |  |
| mean ± sd [Range] | 0.21 ± 0.1 [0-0.37] | 0.31 ± 0.1 [0.15-.046] |
| mv | 1 | 1 |
| RPTOR_CpG_3 |  |  |
| mean ± sd [Range] | 0.52 ± 0.15 [0.16-0.74] | 0.66 ± 0.21 [0-1] |
| mv | 1 | 1 |
| Arg |  |  |
| mean ± sd [Range] | 62 ± 15 [24-91] | 49 ± 12 [31-69] |
| mv | 0 | 0 |
| FUT7_CpG_6 |  |  |
| mean ± sd [Range] | 0.15 ± 0.071 [0.04-0.28] | 0.23 ± 0.11 [0.09-0.45] |
| mv | 1 | 2 |
| RPTOR_CpG_1 |  |  |
| mean ± sd [Range] | 0.086 ± 0.058 [0-0.2] | 0.13 ± 0.059 [0.05-0.24] |
| mv | 1 | 1 |
| FUT7_CpG_3 |  |  |
| mean ± sd [Range] | 0.094 ± 0.072 [0-0.22] | 0.2 ± 0.14 [0.03-0.59] |
| mv | 1 | 2 |
| Met |  |  |
| mean ± sd [Range] | 16 ± 2.5 [11-21] | 14 ± 3.5 [8.5-25] |
| mv | 0 | 0 |
| C3DC |  |  |
| mean ± sd [Range] | 0.2 ± 0.055 [0.11-0.28] | 0.26 ± 0.084 [0.12-0.41] |
| mv | 0 | 0 |
| RPTOR_CpG_5 |  |  |
| mean ± sd [Range] | 0.74 ± 0.072 [0.66-0.89] | 0.81 ± 0.086 [0.65-0.96] |
| mv | 1 | 1 |

**Table V: Descriptive statistic cancer vs cancer free. N=Number, SD=Standard Deviation; MV= missing values**

| **Variables** | **Cancer free** | **Cancer** |
| --- | --- | --- |
| N | 171 | 160 |
| miR_409 |  |  |
| mean ± sd [Range] | 30 ± 2.8 [22-38] | 29 ± 3 [22-36] |
| mv | 6 | 2 |
| SLC22A18_CpG_3 |  |  |
| mean ± sd [Range] | 0.17 ± 0.045 [0.06-0.28] | 0.14 ± 0.051 [0.02-0.41] |
| mv | 3 | 4 |
| SLC22A18_CpG_1 |  |  |
| mean ± sd [Range] | 0.24 ± 0.061 [0.07-0.46] | 0.21 ± 0.068 [0.03 -- 0.52] |
| mv | 3 | 4 |
| HYAL2_CpG_2 |  |  |
| mean ± sd [Range] | 0.2 ± 0.051 [0.06-0.36] | 0.17 ± 0.056 [0.02-0.32] |
| mv | 4 | 2 |
| SLC22A18_CpG_6 |  |  |
| mean ± sd [Range] | 0.22 ± 0.065 [0.02-0.37] | 0.19 ± 0.068 [0.01-0.43] |
| mv | 3 | 4 |
| SLC22A18_CpG_4 |  |  |
| mean ± sd [Range] | 0.19 ± 0.071 [0-0.38] | 0.16 ± 0.076 [0-0.49] |
| mv | 4 | 5 |
| HYAL2_CpG_4 |  |  |
| mean ± sd [Range] | 0.51 ± 0.06 [0.32-0.65] | 0.48 ± 0.076 [0-0.7] |
| mv | 4 | 2 |
| miR_200c |  |  |
| mean ± sd [Range] | 28 ± 4 [21-35] | 26 ± 3.4 [21-33] |
| mv | 7 | 3 |
| HYAL2_CpG_3 |  |  |
| mean ± sd [Range] | 0.35 ± 0.06 [0.18-0.5] | 0.32 ± 0.072 [0.05-0.56] |
| mv | 5 | 5 |
| FUT7_CpG_2 |  |  |
| mean ± sd [Range] | 0.18 ± 0.083 [0-0.48] | 0.15 ± 0.083 [0-0.49] |
| mv | 6 | 6 |

**Table VI: Descriptive statistic EC vs controls. N=Number, SD=Standard Deviation, MV=missing values, C3DC=Malonylcarnitine, C2=Acetylcarnitine, C0=Carnitine, C14:1=Tetradecenoylcarnitine**

| **Variables** | **Controls** | **EC** |
| --- | --- | --- |
| N | 157 | 20 |
| C3DC |  |  |
| mean ± sd [Range] | 0.16 ± 0.074 [0-0.4] | 0.26 ± 0.084 [0.12-0.41] |
| mv | 0 | 0 |
| miR_375 |  |  |
| mean ± sd [Range] | 29 ± 2.5 [22-35] | 32 ± 1.7 [28-36] |
| mv | 7 | 7 |
| C2 |  |  |
| mean ± sd [Range] | 6.3 ± 2.6 [2-14] | 9.3 ± 3.1 [4.6-17] |
| mv | 0 | 0 |
| C0P |  |  |
| mean ± sd [Range] | 24 ± 7.7 [12-54] | 32 ± 6.8 [22-48] |
| mv | 0 | 0 |
| miR_652 |  |  |
| mean ± sd [Range] | 28 ± 2.6 [22-34] | 31 ± 1.9 [27-36] |
| mv | 6 | 0 |
| miR_320b |  |  |
| mean ± sd [Range] | 25 ± 2 [21-31] | 27 ± 0.84 [24-28] |
| mv | 6 | 0 |
| RAPSN_CpG_6 |  |  |
| mean ± sd [Range] | 0.55 ± 0.15 [0.14-0.93] | 0.39 ± 0.17 [0.06-0.77] |
| mv | 6 | 1 |
| S100P_CpG_2_3 |  |  |
| mean ± sd [Range] | 0.63 ± 0.062 [0.47-0.8] | 0.57 ± 0.049 [0.5-0.68] |
| mv | 6 | 1 |
| miR_200c |  |  |
| mean ± sd [Range] | 28 ± 3.9 [21-35] | 24 ± 0.65 [22-25] |
| mv | 7 | 0 |
| C14:1 |  |  |
| mean ± sd [Range] | 0.076 ± 0.039 [0.01-0.22] | 0.11 ± 0.035 [0.05-0.17] |
| mv | 0 | 0 |
